# Dietary Quality Along a Plant-Based Continuum: The Role of Added Salt, Oil, and Sugar in Chronic Disease Risk

**DOI:** 10.3390/nu18152526

**Published:** 2026-08-04

**Authors:** David M. Goldman, Matthew Nagra, Alan C. Goldhamer, Toshia R. Myers

**Affiliations:** 1 Department of Public Health, University of Helsinki, 00014 Helsinki, Finland; 2 Department of Research and Development, Metabite, Inc., New York, NY 10036, USA; 3 Department of Family Practice, University of British Columbia, Vancouver, BC V6T 1Z3, Canada; matthew.nagra@ubc.ca; 4 TrueNorth Health Center, Santa Rosa, CA 95404, USA; dracg@truenorthhealth.com; 5 TrueNorth Health Foundation, Santa Rosa, CA 95404, USA; drmyers@truenorthhealth.org

**Keywords:** whole-food plant-based diet, plant-based dietary pattern, cardiovascular disease, type 2 diabetes, LDL cholesterol, blood pressure, passive overconsumption, dietary sodium

## Abstract

Chronic noncommunicable diseases, including cardiovascular disease (CVD), type 2 diabetes (T2D), and cancer, constitute leading causes of global mortality, with suboptimal diet identified as a primary modifiable risk factor. Plant-based dietary patterns (PBDPs) can be conceptualized along a continuum of quality, with whole-food PBDPs consistently linked to lower risks of CVD, T2D, and all-cause mortality, though much of this evidence derives from studies of vegan or healthy plant-based dietary index patterns rather than whole-plant-food diets directly. Evidence further indicates that minimizing or eliminating added salt, oil, and sugar (SOS) within whole-food PBDPs may provide additional cardiometabolic benefits, primarily by favorably modifying three graded intermediate biomarkers: low-density lipoprotein cholesterol (LDL-C), blood pressure (BP), and adiposity. Dietary sodium increases BP in a linear, dose-dependent manner and enhances food palatability, potentially promoting passive overconsumption and increased adiposity. Although added oil is preferable to saturated fat for LDL-C reduction, preliminary evidence suggests that adding olive oil to an already oil-restricted whole-food PBDP may reduce its LDL-C and body-weight benefits; these findings are hypothesis-generating and require confirmation in larger, longer-term trials. Whole-food fat sources such as nuts and seeds may offer cardiovascular benefits at least equivalent to those of oils, without this attenuating effect. Added sugar promotes excess caloric intake, contributing to increased adiposity, and may impair insulin sensitivity; these effects are most clearly and consistently established for sugar-sweetened beverages (SSBs), whereas the evidence for solid added sugars is weaker and may depend more on excess energy intake and broader dietary context. This narrative review synthesizes evidence from systematic reviews, meta-analyses, Mendelian randomization studies, and key clinical trials to evaluate whether whole-food PBDPs minimizing or eliminating SOS may provide incremental benefit for chronic disease risk reduction and management compared to whole-food PBDPs that include these additives. Direct long-term randomized evidence comparing SOS-restricted to SOS-inclusive whole-food PBDPs remains limited, and the possible incremental benefit of SOS restriction within an already health-promoting dietary pattern warrants further investigation. Complete SOS elimination has not yet been shown to provide incremental benefit beyond a high-quality PBDP, and any benefit is likely greatest in individuals with hypertension, obesity, elevated cardiometabolic risk, or high baseline SOS intake.

## 1. Introduction

Poor diet is a leading modifiable contributor to global mortality, responsible for an estimated 10.9 million deaths annually and a primary driver of noncommunicable chronic disease burden, including cardiovascular disease (CVD), type 2 diabetes (T2D), and cancer [1,2,3]. High sodium intake, low whole grain intake, and low fruit intake together are estimated to account for more than half of all diet-related adult deaths globally [1]. Randomized trials and prospective cohort data consistently demonstrate that plant-based dietary patterns (PBDPs) are associated with substantially reduced chronic disease risk factors [4,5], and the cardiometabolic benefits of PBDPs are among the most consistently replicated findings in nutritional epidemiology: large meta-analyses associate higher adherence to PBDPs with lower risks of CVD, cardiovascular mortality, coronary heart disease, T2D, cancer, and all-cause mortality [5,6,7,8,9]. Proposed mechanisms include increased intake of dietary fiber, unsaturated fat, antioxidants, and phytochemicals; reduced saturated fat and cholesterol exposure; and beneficial effects on the gut microbiome and systemic inflammation [9,10,11].

**Figure 1:**
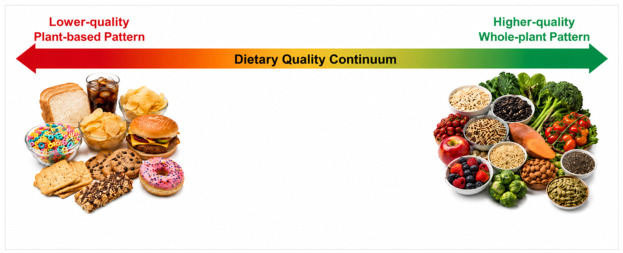
Plant-based dietary quality continuum. Plant-based dietary patterns vary in quality according to the proportion of intact, minimally processed plant foods. Higher-quality patterns emphasize whole grains, legumes, fruits, vegetables, nuts, and seeds, whereas lower-quality patterns contain more refined grains, sugar-sweetened beverages, and processed plant-derived foods. Added salt, extracted oils, and refined sugars may contribute to this quality gradient but are evaluated separately in this review.

These benefits are not uniform across all PBDPs. PBDPs exist along a dietary quality continuum such that higher-quality diets include more minimally processed whole grains, fruits, vegetables, legumes, nuts, and seeds, whereas lower-quality diets include more refined grains, added sugars, and processed plant-derived foods [12,13]. Cardiometabolic outcomes appear to vary along this gradient such that higher-quality PBDPs are associated with a reduction in T2D risk [8], while lower-quality PBDPs are associated with increased risks of T2D, CVD, cancer, and all-cause mortality [5]. A longitudinal analysis of initially healthy adults found that higher adherence to healthy PBDPs was associated with lower BP, fasting glucose, triglycerides, and total-to-high-density lipoprotein-cholesterol ratio, whereas an unhealthy plant-based index showed no such associations [14]. Similarly, a cross-sectional analysis of National Health and Nutrition Examination Survey data found adherence to a whole-food PBDP significantly associated with lower waist circumference, reduced hyperglycemia, lower hypertension prevalence, and improved high-density lipoprotein cholesterol (Figure 1) [13].

One way food quality and processing may shape cardiometabolic outcomes is by altering food matrix integrity, defined here as the structural and biochemical context in which nutrients are delivered. As foods move from minimally processed to highly processed, this context is progressively altered and may change how nutrients function physiologically [15,16]. The physiological effects of matrix disruption may vary in magnitude and direction depending on the nutrient or combination of nutrients delivered, the degree of processing, the amount consumed, and the broader dietary context. Added salt, extracted plant oils, and refined sugars (SOS) are especially relevant because they deliver sodium, fats, and carbohydrates in concentrated forms outside the structure of intact whole foods. Diets high in added salt are associated with elevated BP and increased risks of cardiovascular disease and stroke in a dose-dependent manner [17,18]; diets high in added sugar, particularly sugar-sweetened beverages (SSBs), are associated with increased risks of type 2 diabetes, cardiovascular disease, and all-cause mortality [19,20]; and preliminary evidence suggests that replacing extracted plant oils with intact fat-rich whole foods may produce more favorable effects on LDL-C within whole-food PBDPs [21]. Whether food matrix disruption itself contributes to these effects beyond nutrient quantity alone remains an area of investigation.

To the extent that food matrix integrity contributes to the cardiometabolic gradient described above, exclusively whole-plant-food, SOS-free dietary patterns can be conceptualized as occupying the highest-quality end of this continuum. This dietary pattern has been implemented in residential clinical programs for decades [22,23,24,25], but no studies have isolated the independent contribution of SOS restriction within whole-plant-food dietary patterns. Notably, this clinical-program evidence derives largely from case reports and uncontrolled observations conducted in medically supervised, residential settings, often incorporating prolonged water-only fasting; such reports illustrate feasibility but cannot, on their own, establish an independent effect of SOS restriction in free-living populations. The primary objective of this review is to address this gap through a mechanistic, hypothesis-generating synthesis of how added salt, extracted oils, and refined sugars may influence cardiometabolic risk within whole-plant-food dietary patterns. The synthesis is organized around three established cardiometabolic biomarkers: LDL cholesterol, BP, and central adiposity, each of which confers graded cardiovascular risk when elevated [26,27,28,29]. Each SOS component is evaluated for its potential influence on one or more of these biomarkers. BP is proposed as a point of mechanistic convergence across the salt and sugar components, and palatability-driven passive overconsumption as a shared pathway contributing to central adiposity. The review also identifies research priorities for testing whether SOS restriction provides incremental cardiometabolic benefit within whole-plant-food dietary patterns and in which populations such benefit is most likely.

## 2. Materials and Methods

This narrative review synthesized evidence on PBDPs, SOS restriction and elimination, and chronic disease outcomes. Systematic reviews and meta-analyses were prioritized, supplemented by key randomized controlled trials (RCTs), Mendelian randomization analyses, and clinical program data where meta-analytic evidence was unavailable. A narrative rather than systematic approach was adopted because the primary comparison of interest has not been directly tested and the underlying evidence is heterogeneous in design and not amenable to quantitative pooling. PubMed was searched from inception through March 2026 using terms including “plant-based diet,” “whole food plant-based,” “cardiovascular disease,” “LDL cholesterol,” “dietary sodium,” “blood pressure,” “body weight,” “added sugar,” “dietary fat,” “food matrix,” “passive overconsumption,” and “chronic disease prevention,” with forward and backward citation tracing. PubMed was used as the primary source given the mechanistic, hypothesis-generating aim of this narrative synthesis, with extensive forward and backward citation tracing used to capture relevant records indexed elsewhere. Studies were restricted to adult human populations in peer-reviewed journals. Articles were prioritized for inclusion when they directly addressed plant-based dietary pattern quality, individual SOS components, or one of the three intermediate biomarkers examined here (LDL-C, BP, and adiposity) in adult populations; non-English-language articles, animal studies, and case reports outside the clinical-program literature were excluded. Because exact counts of records identified and screened were not tracked, the approximate scope of coverage reflects the search terms and citation-tracing strategy described above rather than an exhaustive accounting of the literature, and this review should be considered hypothesis-generating rather than a systematic synthesis. Given the narrative design, formal risk of bias assessment and Preferred Reporting Items for Systematic Reviews and Meta-Analyses flow documentation were not performed; instead, convergence across independent study designs was prioritized to strengthen causal inference, and certainty of evidence is noted where source reviews applied Grading of Recommendations Assessment, Development and Evaluation or equivalent frameworks.

The term SOS, referring to added salt, oil, and sugar, originates from clinical practice at TrueNorth Health Center. This dietary construct has not been formally operationalized. Proposed operational definitions and reporting criteria for SOS exposure, intended for research use, are summarized in Table 1. In this review, added salt refers to sodium chloride and sodium-containing seasonings added during manufacturing, preparation, or at the table, excluding sodium intrinsic to intact foods; extracted oils refer to fats separated from their original whole-food matrix, including oils used in cooking or frying and oils added to foods; and refined sugars refer to sugars added during manufacturing, preparation, or at the table, together with sugars in honey, syrups, fruit juices, and concentrates and those in baked and confectionery products, but excluding intrinsic sugars in intact fruits and vegetables. The term “refined” is used here to denote removal of a constituent from its intact food matrix; this differs from the NOVA processing classification, because refined ingredients may span several NOVA groups, whereas “ultra-processed” corresponds most closely to NOVA group 4 [30]. For the purposes of this review, “free of added” (or “SOS-free”) denotes complete exclusion of added salt, oil, or sugar, and “restricted” is used more broadly to denote any meaningful reduction in one or more SOS components, with the specific degree of reduction varying across the studies reviewed. The primary comparison of interest, SOS-restricted versus SOS-inclusive whole-plant-food dietary patterns, has not been directly examined in long-term RCTs. Moreover, much of the existing restriction literature evaluates reductions in individual nutrients against variable dietary backgrounds and thus was not designed to address the whole-food substitution question posed here. Consequently, a narrative approach was used to synthesize epidemiological, clinical, and mechanistic evidence relevant to this question.

## 3. LDL Cholesterol and the Role of Extracted Plant Oils

### 3.1. LDL-C and PBDPs

Among cardiometabolic biomarkers, LDL-C has one of the most consistently established causal relationships with atherosclerotic cardiovascular disease (ASCVD), supported by evidence from Mendelian randomization, prospective cohort studies, and RCTs [26]. Risk reflects both the magnitude and duration of LDL-C exposure, with lifelong lower LDL-C conferring substantially greater protection than LDL-C lowering later in life [26,31]. Subclinical atherosclerosis has been detected across all LDL-C strata within the conventionally normal range, with no risk-free threshold identified in a cohort of 1779 asymptomatic adults [32]. Observations that very low LDL-C is associated with adverse outcomes are generally interpreted as reflecting reverse causation or underlying illness rather than a direct harmful effect of low LDL-C itself [26].

Diet is an important modifiable determinant of LDL-C, and PBDPs consistently demonstrate significant reductions in LDL-C. Across 27 RCTs, PBDPs lowered LDL-C by an average of approximately 12 mg/dL (95% CI: 7–15 mg/dL; *p* < 0.001) compared to omnivorous diets [33]. These effects are mediated in part through displacement of saturated fat, increased dietary fiber and plant sterols inhibiting cholesterol absorption, and viscous fiber reducing bile acid reabsorption [33,34]. The Portfolio dietary pattern, incorporating plant protein, viscous fiber, nuts, and plant sterols, achieves LDL-C reductions of approximately 28 mg/dL beyond those observed with a low-saturated-fat diet [34]. Notably, 45 g per day of monounsaturated fats have been added as a fifth pillar of the Portfolio diet [35], which, when added predominantly in the form of high-oleic sunflower oil, did not impede the effects on LDL-C and further lowered the total cholesterol:HDL-C ratio and C-reactive protein levels, while raising HDL-C [36]. However, the clinical significance of reducing the total cholesterol:HDL-C ratio by raising HDL-C is uncertain: Mendelian randomization and pharmacologic trials indicate that raising HDL-C does not reliably reduce cardiovascular risk, so HDL-C is best regarded as a risk marker rather than a causal treatment target [37].

### 3.2. LDL-C and Extracted Plant Oils Within Whole-Plant-Food Dietary Patterns

Research on dietary fat has largely focused on the relative cardiometabolic effects of saturated versus unsaturated fats, with evidence supporting that substituting saturated fats with polyunsaturated fats reduces CVD risk by approximately 30% [38]. Within PBDPs, however, saturated fat is already substantially reduced [39,40,41], and the relevant question shifts to include delivery context. Fat in intact plant foods is delivered within a structural matrix of fiber, protein, water, micronutrients, and other bioactive compounds that may modulate lipid metabolism, satiety, and dietary compensation [42,43,44], whereas extracted plant oils concentrate the lipid fraction while largely removing this matrix. Whether consuming fat as extracted plant oils versus intact fat-rich plant foods independently influences LDL-C remains an open question. Limited data suggest the effects may vary depending on the foods and oils being compared. Rather than a single overarching effect, outcomes likely depend on the comparator (the food or fat that oil replaces, or that replaces oil), the food matrix, the fatty acid composition of the fat source, and overall energy balance. Intactness (whole-food versus extracted delivery) is therefore conceptually distinct from fatty acid composition, and intact plant foods relatively high in saturated fat warrant separate consideration.

In a 6-week randomized crossover trial, replacing half of participants’ habitual fat intake with either whole almonds or almond oil resulted in comparable reductions in LDL-C [45]. In contrast, both whole avocado and flaxseeds yielded more significant LDL-C reductions than fatty acid-matched oils [46,47]. Interestingly, when not compared directly, the effect sizes for LDL-C reduction are typically larger for PUFA-rich oils than for nuts in clinical trials [48,49]. This finding may be due to differing doses and comparators. PUFA-rich oils are typically compared with saturated fats in clinical trials, whereas nuts may be compared to grains, which may not result in as substantial a difference in LDL-C. Furthermore, no controlled trials have directly compared extracted plant oils with intact fat-rich plant foods for cardiometabolic outcomes within a whole-plant-food dietary context.

The most directly relevant clinical evidence comes from Krenek et al. [21], a randomized crossover trial in which 40 adults at borderline to high ASCVD risk followed an ad libitum whole-plant-food vegan diet in two phases: a high-extra-virgin-olive-oil (EVOO) phase in which extracted oil provided most dietary fat (four tablespoons/day, ≈54 g, which supplied the majority of the 48% of total energy derived from fat in this phase), and a low-EVOO phase in which fat was obtained primarily from intact whole-plant foods, including avocados, nuts, seeds, and olives (<1 teaspoon EVOO/day; 32% of total energy from fat, derived predominantly from intact whole foods, with EVOO contributing negligibly). The low-EVOO phase, therefore, provides a reasonable, though imperfect, approximation of the comparison of interest. Both phases improved total cholesterol, LDL-C, apolipoprotein B, glucose, and high-sensitivity C-reactive protein compared with baseline, reflecting the overall benefit of transitioning to a whole-plant-food dietary pattern. LDL-C reductions were numerically greater, though not statistically significantly different, during the low-EVOO phase than the high-EVOO phase (25.5 vs. 16.7 mg/dL; *p* = 0.16). Sequence analyses showed that LDL-C decreased by 12.7 mg/dL when participants transitioned from high to low EVOO (*p* = 0.04) and increased by 15.8 mg/dL when they transitioned from low to high EVOO (*p* = 0.02), with weight loss 0.7 kg greater in the low-EVOO phase (*p* = 0.01). However, significant order effects violate core crossover assumptions, making it difficult to attribute the findings specifically to EVOO intake rather than dietary sequence or carryover effects. These results are therefore best interpreted as hypothesis-generating, pending replication in larger parallel-arm or controlled feeding trials.

Randomized, placebo-controlled trials have also found EVOO to be less effective for LDL-C reduction compared with other plant-based oils, such as sunflower, corn, or soybean oil, which are richer in PUFA, although EVOO retains monounsaturated fatty acids together with phenolic compounds (notably hydroxytyrosol and oleuropein), tocopherols, and squalene that are largely removed from more refined oils and have been associated with antioxidant, anti-inflammatory, and lipid-modulating effects [50,51]. Moreover, observational studies associate higher EVOO consumption with a 16–23% reduction in CVD risk [52,53,54]. Notably, the PREvención con DIeta MEDiterránea (PREDIMED) trial found similar cardiovascular event reductions for both its EVOO-supplemented and nut-supplemented arms compared with a lower-fat control diet [55], and a prospective analysis found no significant difference in total CVD risk between avocado and plant oil consumption [56], suggesting that intact fat-rich plant foods achieve at least equivalent cardiovascular benefit to extracted oils.

An umbrella review of 23 meta-analyses found that 28 g/day of nuts was associated with significant reductions in CVD risk (RR: 0.79; 95% CI: 0.70–0.89) and all-cause mortality (RR: 0.78; 95% CI: 0.72–0.84) [57], with no adverse effect on body weight at doses up to 100 g/day across 86 RCTs (MD: 0.09 kg; high certainty) [58]. Similarly, an umbrella review of 17 systematic reviews investigating olive oil consumption identified beneficial associations with CVD, cancer, type 2 diabetes, and all-cause mortality [59], while a meta-analysis of randomized controlled trials did not identify an adverse effect on adipose tissue mass unless the oil was delivered via capsules, which may bypass some satiety mechanisms [60]. Similar findings have been identified for other liquid plant oils as well; however, the data comparing extracted oils to whole-plant-based foods are limited to a few studies [61]. As such, replication of the Krenek findings, ideally with various fatty acid compositions, is necessary to draw more definitive conclusions regarding the inclusion or exclusion of plant-based oils within a WFPB dietary pattern.

### 3.3. LDL-C, Added Salt, and Refined Sugars

Beyond extracted plant oils, added salt and refined sugars have weaker direct effects on LDL-C, though each may exert a secondary influence through compensatory or metabolic pathways. Sodium reduction itself has no established primary LDL-C pathway, but short-term sodium-reduction trials have observed modest compensatory increases in total cholesterol (approximately 2.5%) and triglycerides (approximately 7%) alongside activation of renin, aldosterone, and catecholamines [62]. Whether similar compensatory responses occur when sodium intake is naturally low within a mineral-rich whole-food dietary pattern remains an open research question. Refined sugars also lack a clear direct effect on LDL-C, though excess fructose, particularly in liquid form or at hypercaloric doses, promotes hepatic de novo lipogenesis, increasing triglycerides and potentially altering LDL particle composition [63,64]. These mechanisms are supported by controlled feeding studies in which fructose doses greater than 100 g per day increased LDL-C by 11.6 mg/dL [65]. One randomized trial in adults with NAFLD reported adverse lipid and glycemic effects following a fruit-rich diet, suggesting that hepatic metabolic status may modify responses to high fruit or fructose exposure [66]. However, the prescribed dose was a minimum of four servings daily, and the intervention group experienced increased BMI, suggesting that weight gain may have partially driven the results. By contrast, in a controlled feeding study using very high intakes of whole fruit, vegetables, and nuts, there were no adverse effects on insulin secretion and a substantial LDL-C reduction despite high total carbohydrate and fruit intake [67], consistent with the matrix-integrity hypothesis that intrinsic sugars within intact whole foods behave differently from refined sugars delivered outside this context.

## 4. Blood Pressure and the Role of Added Salt

### 4.1. BP and PBDPs

BP has a continuous, graded relationship with cardiovascular risk across clinically relevant ranges. In a meta-analysis of 123 studies involving 613,815 participants, each 10-mmHg reduction in systolic blood pressure (SBP) significantly decreased the risk of coronary heart disease (RR: 0.83; 95% CI: 0.78–0.88) and stroke (RR: 0.73; 95% CI: 0.68–0.77), with benefits observed across baseline BP categories, including SBP below 130 mmHg [68]. Consistent with this graded-risk model, in a cohort of 1457 adults without traditional cardiovascular risk factors, each 10 mmHg increase in SBP from as low as 90 mmHg was associated with a 53% higher risk of ASCVD, and SBP of 120–129 mmHg was associated with approximately 4.6 times the risk of those with 90–99 mmHg [69]. Apparent J-shaped associations between BP and adverse outcomes in observational data are most likely explained by reverse causation, whereby serious illness lowers BP rather than low BP causing harm [68,70].

PBDPs reduce BP across controlled intervention trials, with effects observed for both systolic and diastolic BP across a range of PBDPs from vegan to less restrictive approaches [71,72]. Proposed mechanisms include increased potassium intake, dietary nitrates, and bioactive phytochemicals such as polyphenols that may enhance vasodilation, improve endothelial function, and reduce oxidative stress [71,72]. Evidence for a BP-specific quality gradient is emerging, with higher adherence to healthy PBDPs associated with lower BP, whereas unhealthy PBDPs showed no such association [14].

### 4.2. BP and Added Salt Within Whole-Plant-Food Dietary Patterns

Sodium in intact plant foods is delivered with potassium, magnesium, and other minerals that may modulate its vascular and renal effects [73,74]. Added salt differs substantially, providing sodium in concentrated form outside this mineral balance and structural context [15,16]. Beyond raising blood pressure, high sodium intake, together with its accompanying chloride, has been linked to blood-pressure-independent vascular effects, including impaired nitric-oxide-mediated endothelial function, increased oxidative stress, arterial stiffening, and disruption of the endothelial glycocalyx [75]. Most sodium-reduction trials examine the effects of lowering added salt against variable dietary backgrounds and do not isolate the physiological effects of sodium within intact whole-plant-food dietary patterns.

Sodium reduction lowers BP in a dose-dependent and nearly linear manner across a broad range of intake, with no clear threshold below which BP benefit disappeared [27]. A dose–response meta-analysis of 85 RCTs demonstrated this linear association across a range of 0.4 to 7.6 g per day in both normotensive and hypertensive individuals, with each reduction of approximately 1150 mg corresponding to a mean SBP decrease of 1.10 mmHg (95% CI: 0.66–1.54); reductions down to 400 mg per day remain associated with lower BP [27,76]. An umbrella review provided moderate-to-high certainty evidence that lower sodium intake reduces BP, cardiovascular events, and all-cause mortality [18]. Apparent J-shaped associations between sodium intake and cardiovascular outcomes in some observational studies are most likely attributed to methodological limitations, including spot urine estimation, residual confounding, and reverse causation from pre-existing illness [27,76,77]. Studies using stronger sodium-assessment methods, particularly repeated 24 h urine collections, more consistently support a direct relationship between sodium intake, BP, and cardiovascular risk [76,77].

Although BP responsiveness varies among individuals, with consistently greater effects in those with hypertension and in older adults [78,79,80], sodium reduction remains a fundamental aspect of BP management across populations [18,27]. However, the incremental BP benefit of eliminating added salt within an exclusively whole-plant-food dietary pattern remains poorly characterized. Such patterns are already naturally low in sodium and rich in potassium and magnesium, and the magnitude of additional BP reduction may differ from that observed in sodium-reduction trials conducted against mixed dietary backgrounds. This represents a specific priority for comparative research. Because iodized table salt is a major dietary source of iodine, excluding added salt may reduce iodine intake; adequate iodine should therefore be ensured through appropriate alternative sources, such as iodine-containing foods or supplementation, particularly in individuals following whole-plant-food, SOS-free patterns [81].

Culinary herbs and spices provide a practical approach to maintaining palatability when added salt is reduced; experimental evidence indicates that sodium reductions of 30 to 50 percent can be achieved through the inclusion of herbs and spices without compromising acceptability [82,83]. Moreover, sustaining a low-sodium dietary pattern is associated with adaptation of salt taste preference toward lower sodium concentrations [84,85].

## 5. Central Adiposity and Passive Overconsumption

### 5.1. Central Adiposity and PBDPs

Excess adiposity increases cardiometabolic risk through insulin resistance, dyslipidemia, and elevated BP [29,86,87,88]. Within the overweight and obese BMI range, higher BMI correlates with a progressively greater risk of CVD, T2D, and certain cancers [87,89]. Although BMI is widely used, central adiposity, particularly visceral adipose tissue (VAT), more reliably captures cardiometabolic risk across diverse body compositions and ethnicities [87]. VAT is metabolically active, releasing free fatty acids into the portal circulation and secreting pro-inflammatory adipokines that contribute to insulin resistance, dyslipidemia, systemic inflammation, and RAAS activation [86]. Elevated VAT can occur in individuals with normal BMI, and normal-weight central obesity is associated with greater cardiovascular and all-cause mortality risk than general obesity without central adiposity [90]. Waist circumference, therefore, provides a more complete picture of cardiometabolic risk than BMI alone, particularly in individuals with normal weight.

PBDPs are associated with significant reductions in BMI, with pooled estimates ranging from −0.90 to −1.38 kg/m^2^ [29,88,91,92], and significant reductions in waist circumference of 2.20–2.41 cm [91,92] across multiple meta-analyses of RCTs. These reductions are likely attributable to decreased energy density from lower fat and higher water and fiber content, which enhances satiety without requiring explicit energy restriction [29,88,91]. Evidence for a central-adiposity-specific quality gradient is emerging, with adherence to a whole-food PBDP associated with lower waist circumference, whereas less healthy PBDPs showed no comparable benefit [13].

### 5.2. SOS, Palatability, and Passive Overconsumption Within Whole-Plant-Food Dietary Patterns

Added salt, extracted plant oils, and refined sugars have not been directly characterized for their independent effects on central adiposity within whole-plant-food dietary patterns, but each may contribute to passive overconsumption by altering palatability, satiety, or energy compensation mechanisms that normally limit caloric intake from intact whole-plant foods [42,93,94]. Hyperpalatable foods are characterized by threshold-concentration combinations of fat–sodium, fat–sugar, and carbohydrate–sodium and are associated with increased ad libitum energy intake [94,95]. Although naturally occurring combinations of sodium, fat, carbohydrate, and sugar in intact foods may sometimes reach hyperpalatable thresholds, added salt, extracted oils, and refined sugars concentrate these components outside the structural and biochemical context of whole foods, potentially making threshold-level combinations easier to achieve. Experimental evidence further indicates that specific nutrient combinations, particularly fat and carbohydrate, can produce supra-additive effects on food reward, supporting the plausibility that SOS combinations may increase reward value and contribute to positive energy balance over time [94,96,97]. The evidence reviewed is primarily from generally healthy or overweight adults and may not apply to individuals with increased caloric requirements.

Added salt appears to contribute to palatability-driven passive overconsumption primarily in combination with dietary fat or carbohydrates. In studies of salt-fat combinations, salt had a stronger effect than fat on pleasantness ratings of savory fatty foods and, in a separate ad libitum meal study, salt increased food and energy intake and bypassed fat-mediated satiation in individuals sensitive to fat taste [98,99]. Hyperpalatable carbohydrate–sodium combinations have been associated with greater weight and body fat gain at one-year follow-up [94], while short-term free-living data link hyperpalatable fat–sodium combinations to greater within-meal energy intake and eating despite satiation [97]. Whole, unprocessed plant foods are naturally low in sodium, typically providing well below 1 g per day in the absence of added salt [100]; within whole-plant-food dietary patterns, the absence of added salt removes a primary driver of hyperpalatable sodium-containing combinations and may reduce the risk of palatability-driven passive overconsumption, though this has not been directly studied in long-term trials.

Extracted plant oils are highly energy-dense and lack the fiber, protein, water, and structural matrix of intact fat-rich foods that may contribute to satiety and energy compensation [43,93]. Compared with whole nut consumption, extracted nut oils elicit substantially weaker dietary compensation in individuals with overweight, as low as 4% versus 55–75% for whole nuts, suggesting that food matrix integrity contributes to appetite regulation beyond lipid content alone in those prone to overconsumption [42,44]. In lean individuals, compensation responses to extracted oils and whole nuts appear more comparable [42], consistent with evidence that extracted oils can stimulate satiety signaling and subjective satiety in generally healthy individuals [101,102]. As noted above, fat-rich meals have been shown to increase energy intake independently, and hyperpalatable fat–sodium combinations further compound this effect; separately, high-fat and high-sugar combinations have been shown to independently predict overeating and weight gain beyond energy density alone [97,99,103]. Whether these compensation dynamics apply within a whole-plant-food dietary context, where oils would be added to an otherwise intact food matrix, has not been directly studied.

Sugars within intact whole-plant foods are delivered within cellular structures that modulate absorption, satiety signaling, and metabolic response [16,63,104]. Whole fruit produces greater satiety and lower subsequent energy intake than applesauce or fruit juice, suggesting that physical food structure, not fiber content alone, contributes to the satiating effects of intrinsic sugars [16,105]. The independent contribution of solid refined sugar to central adiposity through passive overconsumption is more difficult to establish; when liquid and solid sugar sources are disaggregated, the strongest signal is driven primarily by SSBs rather than solid refined sugar [106]. Solid refined sugars may be most relevant to passive overconsumption when combined with extracted oils, added salt, or refined carbohydrates in hyperpalatable foods. High-fat and high-simple-sugar combinations have been linked to overeating and weight gain; fat-carbohydrate combinations can increase food reward, and carbohydrate–sodium combinations have been associated with greater weight and body fat gain [94,96,103]. Whether eliminating refined sugar provides additional benefit within an already low-energy-density whole-plant-food dietary pattern remains an open question.

### 5.3. Liquid Sugar: A Distinct Direct Pathway

**Figure 2:**
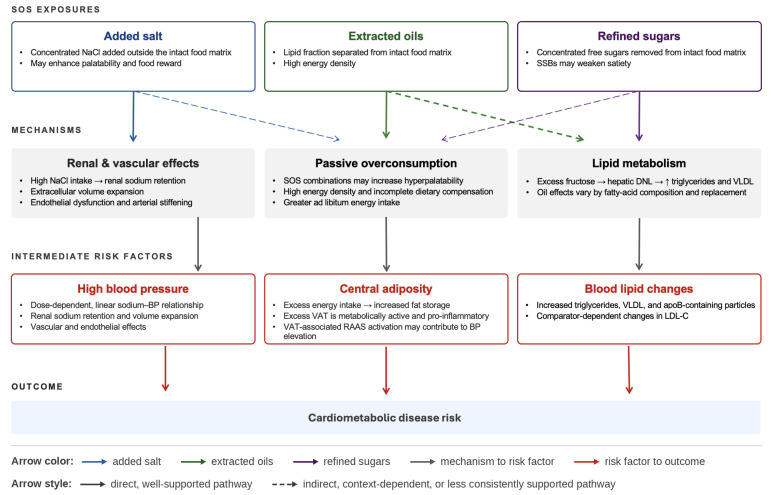
Proposed pathways linking SOS components to cardiometabolic risk. SOS, added salt, extracted oils, and refined sugars; SSB, sugar-sweetened beverage; RAAS, renin–angiotensin–aldosterone system; VAT, visceral adipose tissue; DNL, de novo lipogenesis; VLDL, very-low-density lipoprotein; LDL-C, low-density lipoprotein cholesterol; apoB, apolipoprotein B; BP, blood pressure. Added salt increases cardiometabolic risk through renal sodium retention, extracellular volume expansion, and vascular and endothelial effects, contributing to higher blood pressure in a dose-dependent manner. Across SOS components, hyperpalatable fat–salt, fat–sugar, and carbohydrate–sodium combinations may promote passive overconsumption, excess energy intake, and central adiposity. Excess fructose alters lipid metabolism through hepatic de novo lipogenesis, increasing triglycerides and VLDL, whereas the effects of extracted oils on LDL-C vary according to fatty acid composition and the food or nutrient displaced. Solid arrows indicate direct, well-supported pathways; dashed arrows indicate indirect, context-dependent, or less consistently supported pathways. Most supporting evidence derives from populations consuming mixed dietary backgrounds.

Liquid refined sugar exerts effects beyond palatability alone. Refined or free sugars are removed from their native cellular structure, concentrated, and, in liquid form, delivered outside the broader food matrix of water, fiber, and bioactive compounds that normally modulate absorption, satiety signaling, and metabolic response [16,63,104]. This delivery may bypass physiological satiation mechanisms more readily than solid foods, promoting excess energy intake and adverse metabolic effects [63,64]. Consistent with this, beverage-guidance frameworks have long ranked calorically sweetened beverages among the least preferable sources of dietary fluids [107]. Added sugar intake has been associated with CVD mortality in a dose-dependent manner. In a cohort of 11,733 US adults followed for 14.6 years, consuming 10–25% of calories from refined sugar was associated with a 30% higher risk of CVD mortality (HR: 1.30; 95% CI: 1.09–1.55), rising to a 2.75-fold increased risk at 25% or more (HR: 2.75; 95% CI: 1.40–5.42) [20]. This study did not disaggregate liquid from solid refined sugar sources; however, when sugar sources are examined separately, the strongest and most consistent adverse associations are observed for SSBs. SSB consumption was linked to a 27% higher risk of T2D (RR: 1.27; 95% CI: 1.15–1.41) and a 9% higher CVD risk across 34 studies, as well as an 8% increase in all-cause and CVD mortality per additional daily serving [19,108]. A systematic review with formal certainty assessment found SSBs robustly associated with T2D risk (pooled RR: 1.25; 95% CI: 1.17–1.35; moderate certainty) across 541,288 participants [106]. Beyond bypassing satiation, excess fructose in liquid form or at hypercaloric doses promotes hepatic de novo lipogenesis, increasing triglycerides and contributing to insulin resistance and ectopic fat accumulation [63,64]. Together, these data suggest that sugar-related cardiometabolic risk follows a matrix-integrity gradient, with the strongest evidence for liquid sugars delivered outside the structural context of intact whole-plant foods and weaker evidence for solid refined sugars. Consistent with this gradient, the independent contribution of solid added sugars to cardiometabolic risk is less certain and appears to depend more on excess energy intake and the broader dietary context than does that of SSBs. Whether further restriction of solid refined sugars within an already whole-plant-food dietary pattern provides additional benefit along this gradient remains unknown. Table 2 and Figure 2 summarize these proposed mechanisms linking each SOS component to LDL-C, BP, and central adiposity, together with representative references and an indication of evidence strength.

## 6. Research Priorities

There is biological plausibility for SOS restriction, but no trials have directly compared SOS-restricted and SOS-inclusive whole-plant-food dietary patterns. Therefore, the central unanswered question is whether a whole-plant-food dietary pattern free of added salt, extracted oils, and refined sugar provides additional cardiometabolic benefit compared with a whole-plant-food dietary pattern that includes these components. The highest-priority studies are direct parallel-arm trials, with LDL-C and/or apolipoprotein B, 24 h ambulatory BP, and waist circumference as primary endpoints, that are conducted within otherwise comparable whole-plant-food dietary patterns. Trials should use validated dietary instruments to quantify SOS exposure and adherence, supplemented by available biomarkers such as repeated 24 h urinary sodium collections for sodium assessment. Patient-reported outcomes, including satiety, food liking, diet acceptability, and quality of life, should also be incorporated, along with monitoring of total energy intake and key nutrients of concern, including iodine, zinc, vitamin B12, vitamin D, and omega-3 fatty acid status. Component-specific exposure and adherence data should be collected to support evaluation of individual SOS components, pairwise combinations, and full SOS restriction, allowing additive or interactive effects to be evaluated where feasible. These trials will require validated dietary assessment instruments capable of distinguishing added salt from naturally occurring sodium, extracted oils from intact fat-rich whole foods, and refined sugars from intrinsic whole-food carbohydrates.

Component-specific priorities should determine whether the physiological effects of added salt, extracted oils, and refined sugars depend on dietary context, food matrix integrity, and dose. For added salt, key questions include whether the sodium-BP dose–response established in mixed dietary populations holds within patterns already naturally low in sodium, and whether compensatory RAAS activation and lipid changes occur when sodium intake is naturally low within dietary patterns high in potassium and magnesium. For extracted oils, the primary priority is to determine whether replacing oils with intact, fat-rich plant foods reduces LDL-C independently of total fat quantity, using fat-matched parallel-arm designs. For refined sugars, priorities include whether fructose–uric acid signaling and fructose–sodium renal interactions operate at intakes typical of whole-plant-food dietary contexts, and whether eliminating solid refined sugars provides additional benefit within an already low-energy-density dietary pattern. Insulin resistance may also warrant evaluation within these trials, given its relationship to central adiposity and potential responsiveness to refined-sugar reduction.

A related priority is determining whether complete SOS elimination is necessary for all outcomes. Palatability-driven passive overconsumption may be the mechanism for which near-complete elimination is most necessary, whereas effects on LDL-C and BP may depend on dose, baseline clinical characteristics, and dietary context. Evidence indicates that salt taste preference is bidirectionally plastic, shifting over weeks to months of dietary change, and that exposure to combined high-fat, high-sugar foods can alter hedonic response independent of body weight change [84,109,110]. Whether similar palatability resetting occurs within already low-SOS whole-plant-food dietary patterns remains unknown, and future trials should examine whether SOS-free patterns reduce ad libitum energy intake compared with SOS-inclusive patterns when energy density is matched.

The incremental benefit and applicability of SOS restriction are likely to vary by baseline clinical, metabolic, and dietary characteristics. Trials should prospectively stratify participants by hypertensive status, baseline LDL-C, central adiposity, insulin resistance, baseline SOS intake, metabolic phenotype, sodium sensitivity, and energy requirements. Effects may be largest in individuals with established cardiometabolic risk or high baseline SOS intake, whereas the benefit in normotensive, metabolically healthy individuals already consuming a high-quality whole-plant-food diet may be smaller and harder to detect. Whether whole-plant-food, SOS-free dietary patterns can consistently meet the energy and nutrient demands of athletes, pregnant and breastfeeding women, children, and individuals with high caloric requirements warrants specific investigation alongside trials focused on cardiometabolic outcomes.

Addressing these priorities would help establish whether SOS restriction represents a clinically meaningful refinement within an already high-quality whole-plant-food dietary pattern.

## 7. Limitations

This review adopts a narrative design without systematic search and screening procedures; therefore, selection bias cannot be excluded, and citation selection may favor evidence consistent with the potential benefits of SOS restriction and the food matrix framework. The food matrix framework advanced here is hypothesis-generating, as direct evidence that delivery context independently alters physiological outcomes beyond quantity effects remains limited. Moreover, no long-term RCTs have directly examined intact whole-food nutrient delivery compared with extracted or refined forms within whole-food plant-based dietary patterns. As a result, evidence strength varies substantially across the three SOS components and must be interpreted with these constraints.

The sodium–BP relationship is strongly and consistently supported, but whether compensatory RAAS activation and lipid changes observed during imposed sodium restriction occur when sodium intake is naturally low within a mineral-rich whole-food dietary pattern remains unresolved. The oil evidence rests primarily on a single small crossover trial with significant order effects; findings are therefore hypothesis-generating and require replication. Evidence for cardiometabolic effects of solid refined sugar independent of SSBs is weak or absent for most outcomes, and the fructose–uric acid BP pathway is established primarily at supraphysiological doses. The proposed convergence of all three SOS components to palatability-driven passive overconsumption and central adiposity is mechanistically plausible but has not been tested as an integrated hypothesis in human dietary trials.

Much of the dietary-pattern evidence linking overall diet quality with chronic disease outcomes derives from observational studies, in which residual confounding remains possible. Additionally, much of the available evidence derives from populations consuming typical Western dietary patterns with high baseline SOS intake, limiting direct extrapolation to whole-plant-food dietary contexts. Population heterogeneity also remains insufficiently addressed, as few studies directly evaluate whether the effects of SOS restriction differ by baseline nutrient sensitivity, cardiometabolic risk, medication use, or background diet quality. For example, the net cardiometabolic benefit of further SOS restriction in normotensive individuals already consuming a whole-plant-food dietary pattern remains uncertain. Whether any incremental benefit of SOS restriction within an already favorable dietary pattern is detectable in trials of feasible duration and sample size remains unknown.

Finally, long-term palatability, adherence, and sustainability of exclusively whole-plant-food SOS-free dietary patterns have not been rigorously examined, and adherence outcomes are likely context-dependent and influenced by structured support [111,112].

## 8. Conclusions

The evidence reviewed supports a continuum-based model of plant-based dietary quality in which greater emphasis on whole, minimally processed plant foods is associated with more favorable cardiometabolic outcomes. Within this framework, SOS restriction may represent a further refinement, and food matrix integrity offers one mechanistic lens for interpreting this gradient: as foods move from intact to refined forms, the structural and biochemical context of nutrient delivery is altered, and this change in delivery context may influence physiological responses relevant to LDL-C, BP, and central adiposity. BP represents a plausible point of mechanistic convergence for added salt and refined sugars, while palatability-driven passive overconsumption represents a shared pathway through which added salt, extracted oils, and refined sugars may contribute to central adiposity. The framework does not require whole-food delivery to be uniformly superior to extracted forms; rather, it proposes that delivery context is a biologically relevant variable warranting direct investigation.

Clinically, the available evidence supports helping patients move along the dietary quality continuum toward predominantly whole, minimally processed plant foods while reducing added salt, extracted oils, and refined sugars as appropriate to individual goals, risks, and preferences. This framework does not imply that SOS reduction must be absolute; partial reductions within predominantly plant-based diets are also likely to confer meaningful cardiometabolic benefit relative to typical Western dietary patterns. Direct long-term comparisons between SOS-restricted and SOS-inclusive whole-plant-food dietary patterns remain limited, and future controlled feeding and pragmatic comparative trials are needed to determine whether complete SOS restriction provides a measurable incremental benefit beyond an already high-quality whole-plant-food dietary pattern. The strongest expected effects are in individuals with hypertension, elevated cardiometabolic risk, or high baseline SOS intake. Consistent with the evidence gradient described above, the case for reduction is strongest and most consistent for SSBs, whereas the incremental benefit of eliminating solid added sugars within an already whole-plant-food pattern is less certain. Importantly, complete SOS elimination has not yet been proven to confer cardiometabolic benefit beyond that of a high-quality whole-plant-food diet; this remains a question for future trials rather than an established conclusion. At the population level, policy measures such as front-of-package warning labels for products high in sugar, sodium, or saturated fat have been associated with reduced purchases of these nutrients and may complement individual dietary change [113,114].

## Figures and Tables

**Table 1:** Proposed operational definitions and reporting criteria for SOS exposure.

Term	Definition	Examples
SOS-free	No added salt, extracted oils, or refined sugars. Naturally occurring sodium, fat, and intrinsic sugars in intact foods may be present within the overall dietary pattern.	Oats with fruit; lentil soup prepared with salt-free vegetable broth and herbs; salad with avocado and lemon.
SOS-restricted	One or more SOS components limited to a prespecified amount and reported separately.	Oatmeal with measured maple syrup; lentil soup with a measured amount of added salt; salad with measured olive oil.
SOS-inclusive	One or more SOS components included within an otherwise predominantly whole-plant-food pattern.	Oatmeal with brown sugar; lentil soup prepared with high-sodium broth or added salt; salad with oil-based dressing.

SOS, added salt, extracted oils, and refined sugars. Added salt includes sodium chloride and sodium-containing seasonings added during manufacturing, preparation, or at the table; naturally occurring sodium in intact foods is excluded. Refined sugars include sugars added during manufacturing, preparation, or at the table, as well as sugars in honey, syrups, fruit juices, and juice concentrates; intrinsic sugars in intact fruits and vegetables are excluded. Sugar-sweetened beverages contain added caloric sweeteners regardless of sweetener form. Extracted oils are fats separated from their original intact food matrix. SOS-restricted and SOS-inclusive limits should be prespecified, and each component should be reported separately. SOS-inclusive is heterogeneous and should not be treated as a uniform exposure. These categories are proposed for research reporting and have not been clinically validated.

**Table 2:** Proposed mechanisms linking SOS components to cardiometabolic risk factors.

SOS Exposure	Proposed Pathway	Primary Outcome(s)	Evidence	Evidence Strength
Added salt	Sodium and chloride may promote volume expansion, renal sodium retention, and vascular effects.	Blood pressure	Dose-responsive BP effects are well established across mixed dietary populations. The incremental effect within an otherwise naturally low-sodium WFPB diet has not been adequately tested.	Strong overall; indirect for the specific WFPB comparison [17,18]
Added salt in mixed foods	Salt may increase palatability and food reward, particularly in fat–salt and carbohydrate–sodium combinations.	Energy intake; central adiposity	Short-term studies support effects on liking and ad libitum intake. Isolated salt addition within a WFPB pattern has not been directly tested.	Limited to moderate; indirect [94,97,98,99]
Extracted oils	High energy density and loss of intact food structure may weaken dietary compensation in some populations.	Energy intake; central adiposity	Intact nuts may elicit greater compensation than extracted nut oils, though responses vary. Whether oil addition within an otherwise intact WFPB pattern alters long-term intake or adiposity is unknown.	Limited and context-dependent [42,43,44,93,101,102]
Extracted oils versus intact fat-rich foods	Delivery context may affect lipid metabolism; effects depend on fatty acid composition and the food displaced.	LDL-C; ApoB	Direct comparisons are mixed and sparse. Within-WFPB evidence is small and methodologically limited.	Limited; hypothesis-generating [21,45,46,47]
Sugar-sweetened beverages (SSBs)	Limited satiety signaling and rapid delivery may increase energy intake; hypercaloric exposure may promote hepatic de novo lipogenesis.	Central adiposity; insulin resistance; CVD and T2D risk	Prospective and experimental evidence supports adverse associations regardless of sweetener type. Typical studies do not isolate modest SSB exposure within an otherwise WFPB diet.	Moderate to strong overall; indirect for WFPB [19,20,63,64,106,108]
Solid refined sugars	Effects may occur through increased palatability and combinations with fat, refined starch, or sodium.	Energy intake; central adiposity	Independent effects are weaker and less consistent than for SSBs. Direct evidence within WFPB patterns is lacking.	Limited or inconsistent [94,96,103,106]
SOS combinations	Fat–salt, fat–sugar, and carbohydrate–sodium combinations may increase food reward and weaken satiety regulation.	Energy intake; central adiposity	Experimental and observational evidence supports hyperpalatable combinations as a driver of passive overconsumption. This pathway has not been tested in SOS-inclusive versus SOS-free WFPB diets.	Limited to moderate; indirect [94,95,97,98,99,103]

SOS, added salt, extracted oils, and refined sugars; WFPB, whole-food plant-based; LDL-C, low-density lipoprotein cholesterol; ApoB, apolipoprotein B; SSB, sugar-sweetened beverage; CVD, cardiovascular disease; T2D, type 2 diabetes. Evidence levels reflect this narrative synthesis, not formal GRADE ratings, and distinguish general pathway evidence from evidence directly applicable to whole-plant-food dietary contexts. SSBs refer to refined or free sugars consumed in beverage form, regardless of sweetener type.

## Data Availability

No new data were created or analyzed in this review. Data sharing is not applicable to this article.

## Abbreviations

The following abbreviations are used in this manuscript:

ApoB	Apolipoprotein B
ASCVD	Atherosclerotic cardiovascular disease
BP	Blood pressure
CVD	Cardiovascular disease
EVOO	Extra-virgin olive oil
LDL-C	Low-density lipoprotein cholesterol
NOVA	Nova food classification system
PBDP	Plant-based dietary pattern
RAAS	Renin–angiotensin–aldosterone system
RCT	Randomized controlled trial
SOS	Added salt, oil, and sugar
SSB	Sugar-sweetened beverage
T2D	Type 2 diabetes
VAT	Visceral adipose tissue
WFPB	Whole-food plant-based
